# Associations of Biofilm Capacity with Antimicrobial Resistance and Virulence Genes in *Klebsiella pneumoniae* from Chickens in Henan, China, 2023–2025

**DOI:** 10.3390/antibiotics15080767

**Published:** 2026-08-10

**Authors:** Han Zhang, Jiayi Li, Shiqi Deng, Jiani Lei, Liping Gao, Xiangyang Li, Lili Gao, Yuyang Yuan, Hua Wu, Yajun Zhai, Jianhua Liu

**Affiliations:** College of Veterinary Medicine, Henan Agricultural University, Zhengzhou 450046, China; zhanghan.zz@hotmail.com (H.Z.); lijiayiup@163.com (J.L.); 17513303366@163.com (S.D.); leijiani2000@163.com (J.L.); glpzhs@126.com (L.G.); 19273734508@163.con (X.L.); 18364164693@163.com (L.G.); 19838651432@163.com (Y.Y.); huatongzhi66@163.com (H.W.); zyj90518@126.com (Y.Z.)

**Keywords:** chicken, *K. pneumoniae*, multidrug resistance, virulence gene, biofilm

## Abstract

**Background:** *Klebsiella pneumoniae* is an opportunistic zoonotic pathogen that can cause respiratory diseases in chickens. The aim of this study was to investigate the biofilm formation, resistance, and virulence of *K. pneumoniae* isolates from chickens in Henan Province, China, between 2023 and 2025, and to analyze the associations among these characteristics. **Methods:** We identified the isolates through blood agar culture, Gram staining and matrix-assisted laser desorption ionization-time of flight mass spectrometry (MALDI-TOF MS). Hypermucoviscosity phenotype, biofilm-forming ability, antimicrobial resistance and virulence genes were assessed by the string test, crystal violet assay, disk diffusion and polymerase chain reaction (PCR), respectively. **Results:** A total of 102 *K. pneumoniae* isolates were identified. Of these, 81.4% were biofilm-forming strains, and 15.7% exhibited the hypermucoviscosity phenotype. The susceptibility profiles of the *K. pneumoniae* isolates indicated high resistance to penicillins (>90.0%) and low resistance to carbapenems (<10%), with 88.2% identified as multidrug-resistant. The results of virulence gene detection indicated that *luxS* (79.4%), *mrkD* (75.5%), and *uge* (73.5%) were the most prevalent virulence genes, followed by *wabG* (49.0%), *ybtA* (34.3%), and *iucA* (20.6%). The association analysis indicated that biofilm formation was significant association with multidrug-resistant and the carriage of virulence genes. Compared with non–biofilm–forming isolates, biofilm–forming isolates showed significantly higher resistance to ceftiofur and florfenicol, as well as higher detection rates of *luxS*, *mrkD*, and *uge* (*p* < 0.05). **Conclusions:** The data obtained in this study reveal the pathogenic potential and multidrug–resistant characteristics of this pathogen, highlighting the need for surveillance and monitoring.

## 1. Introduction

*Klebsiella pneumoniae* is an important opportunistic pathogen belonging to the family Enterobacteriaceae. This pathogen causes various human infections, including urinary tract, respiratory, and bloodstream infections [1,2]. Data from the China Antimicrobial Resistance Surveillance System in 2021 indicated that *K. pneumoniae* accounted for 13.9% of human clinical isolates, ranking second only to *Escherichia coli*. In addition to human infections, *K. pneumoniae* is widely prevalent in poultry and is a major pathogen responsible for respiratory disease in chickens, with isolation rates ranging from 9.0% to 35.0% [3,4,5]. The pathogenicity of *K. pneumoniae* is attributed to virulence factors that enable it to evade host defenses and establish an infection. Among the virulence factors, particular attention has been paid to mucoviscosity, lipopolysaccharide (LPS), iron uptake systems, and adhesins, which collectively contribute to bacterial survival and pathogenicity in the host [6].

The absence of effective vaccines against *K. pneumoniae* means that clinical control still relies heavily on antimicrobials. Inappropriate antimicrobial use in poultry production accelerates the emergence of resistant bacteria, especially multidrug-resistant (MDR) strains, which poses a serious threat to both animal and human health [7]. An epidemiological investigation of *K. pneumoniae* from broilers in Shandong Province, China, revealed a high MDR rate of 87.9% [8]. This rate was higher than that reported in Hong Kong, China (22.0%) and comparable to that reported in India (85.0%), but slightly lower than that reported from a broiler slaughterhouse in Shandong Province (96.7%) [9,10]. These findings indicate that antimicrobial resistance in *K. pneumoniae* is widespread in poultry production.

*K. pneumoniae* is difficult to eradicate due to its biofilm-forming ability. Biofilms are structured communities composed of bacteria and their secreted extracellular polymeric substances. This structure facilitates bacterial colonization of host tissues and protects the bacteria from host immune responses, leading to chronic and recurrent infections [11]. In addition, the physical barrier and unique microenvironment within biofilms enhance bacterial tolerance to antimicrobial agents, representing an important factor contributing to treatment failure [12]. Overall, biofilm is an important factor influencing the pathogenicity and antimicrobial resistance of *K. pneumoniae*.

Molecular epidemiological studies of chicken derived *K. pneumoniae* have been conducted in several regions of China, including Jiangsu [1], Shandong [8], and Xinjiang [13]. However, most studies have focused on single aspects, such as antimicrobial resistance, virulence factors, or biofilm formation, while analyses examining the associations among these traits remain limited. Henan Province is a major poultry-producing region in China, and its complex farming background may contribute to diverse epidemiological features of *K. pneumoniae*, leading to distinct regional variations. However, epidemiological investigations of chicken derived *K. pneumoniae* in this region are still lacking, especially those addressing the associations among these traits. Given the important role of biofilms in enhancing bacterial resistance and virulence, this study aimed to investigate the association of biofilm formation with antimicrobial resistance and virulence in chicken derived *K. pneumoniae* isolates collected in Henan Province between 2023 and 2025.

## 2. Results

**Figure 1:**
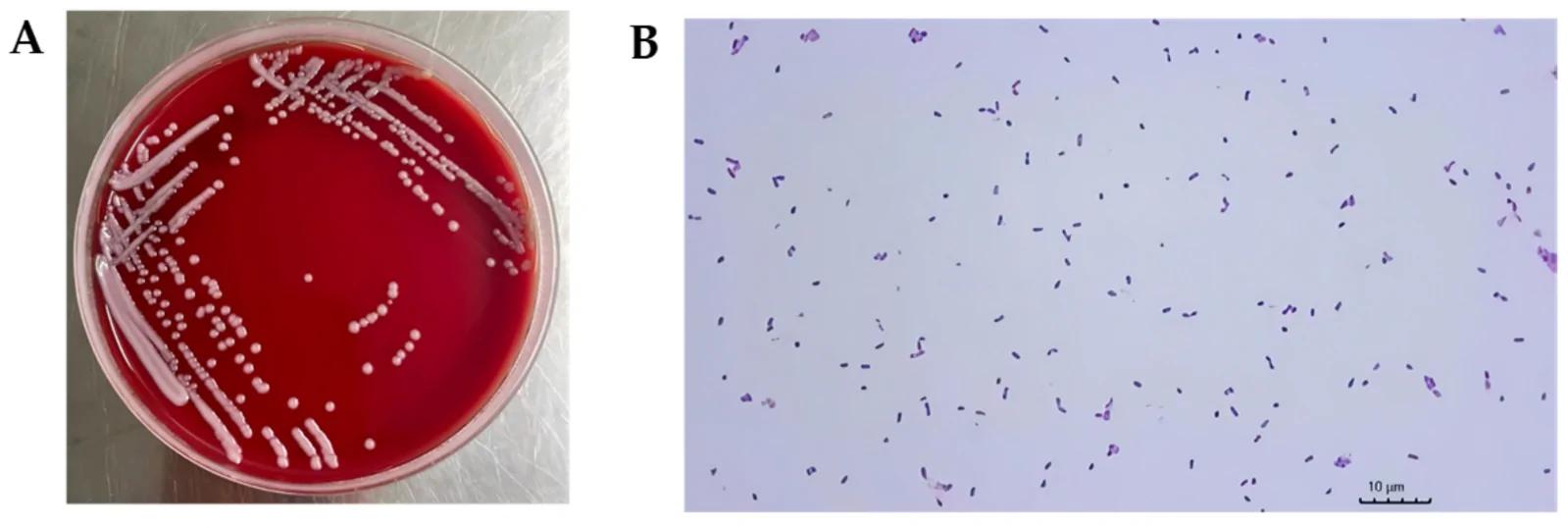
Colony morphology formed and Gram staining. (**A**): Colony morphology of *K. pneumoniae* on blood agar plate. (**B**): Gram staining results of *K. pneumoniae* under a light microscope at ×1000 magnification.

**Figure 2:**
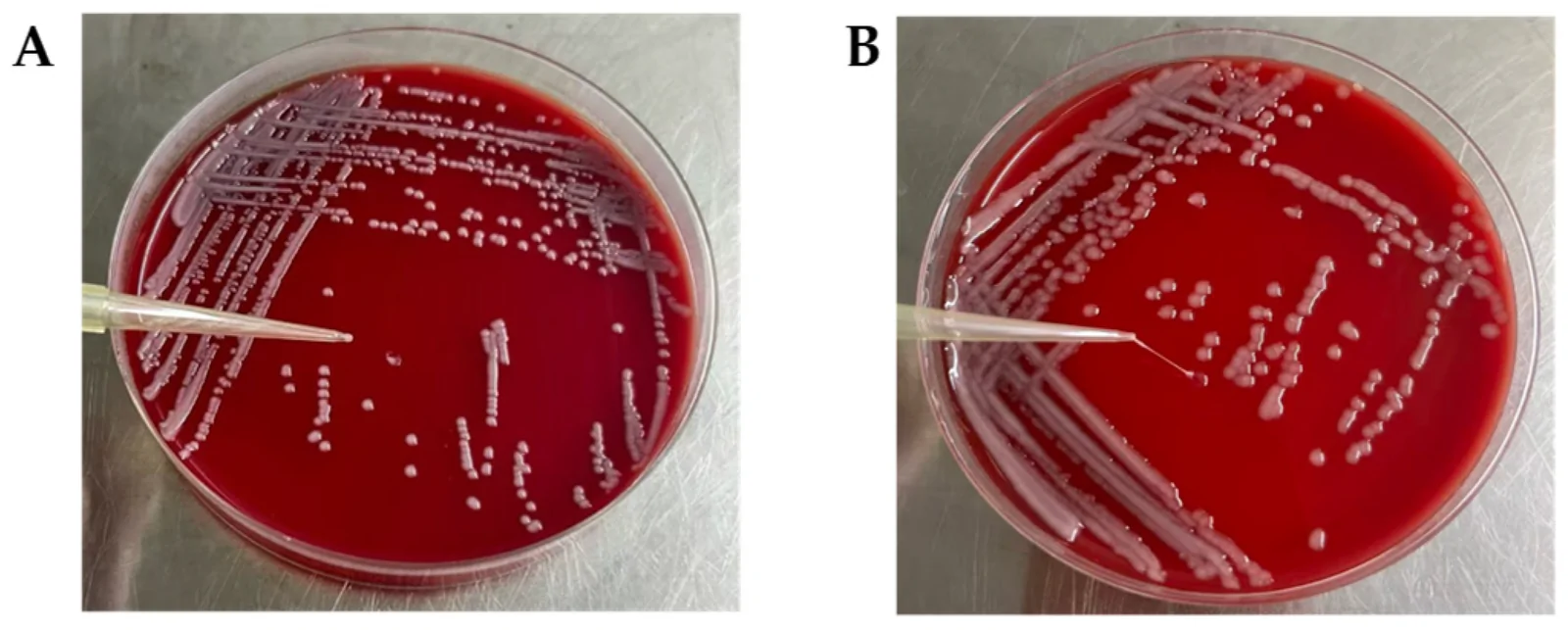
String test for hypermucoviscosity. (**A**): Negative string test for hypermucoviscosity; (**B**): Positive string test for hypermucoviscosity.

The isolates formed round, convex, smooth, milky white colonies on blood agar plates (Figure 1A). Gram staining revealed short, blunt-ended rods occurring singly (Figure 1B). Further identification by MALDI-TOF mass spectrometry confirmed that 102 isolates were *K. pneumoniae.* The hypermucoviscous phenotype was identified in 16 of the isolates (Figure 2).

**Figure 3:**
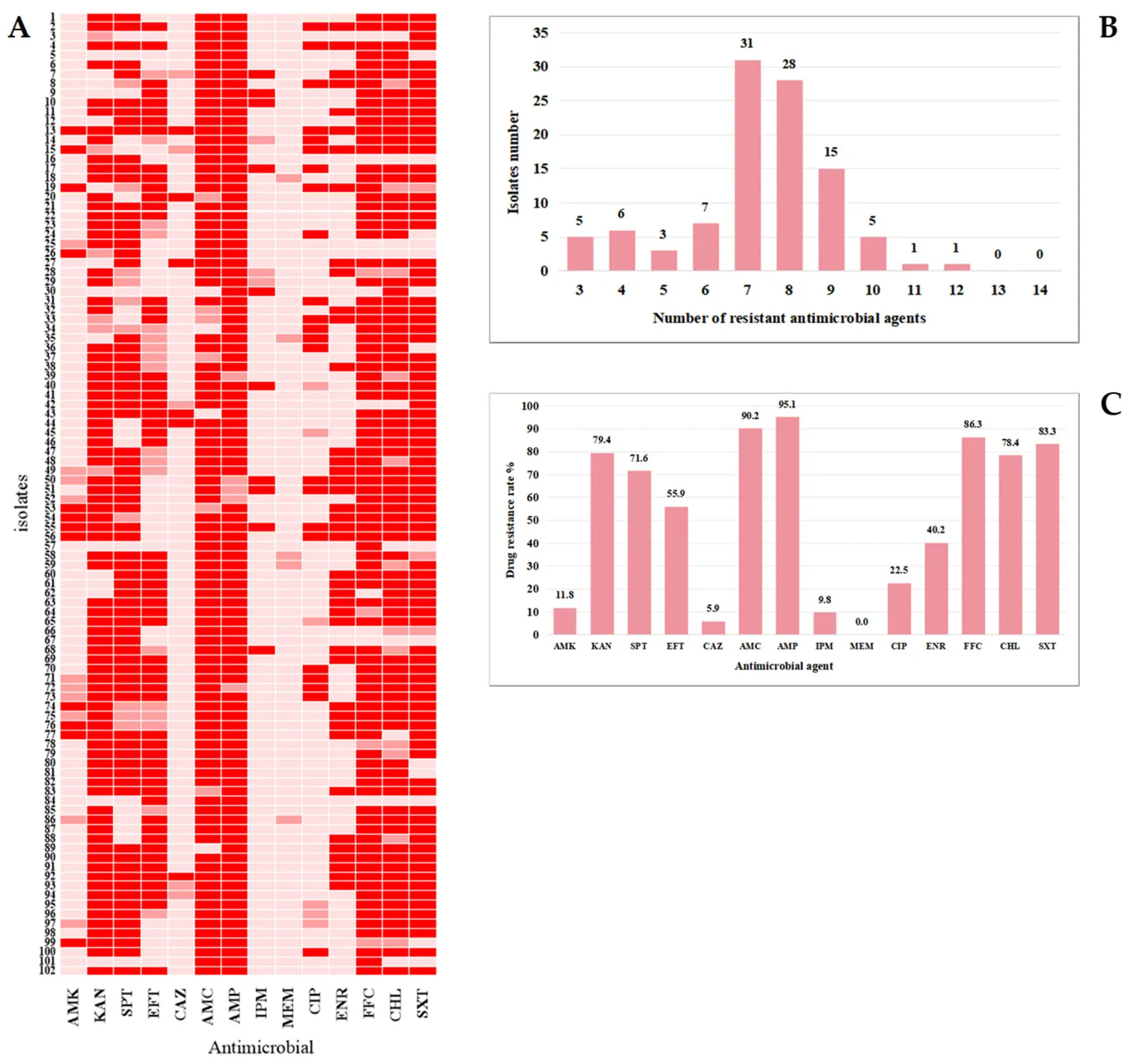
Antimicrobial resistance characteristics of *K. pneumoniae* isolates. (**A**) Heatmap of disk diffusion susceptibility of *K. pneumoniae* to 14 antimicrobial agents, red represents drug resistance, pink represents intermediate, and light pink represents sensitivity. (**B**) Resistance rates to the tested antimicrobials. (**C**) Distribution of isolates according to the number of resistant antimicrobials.

The antimicrobial resistance profile revealed varying degrees of resistance to the 14 tested antimicrobials (Figure 3A). High resistance rates were observed for penicillins, whereas low resistance rates were observed for carbapenems (Figure 3B). Among the isolates, 31 were resistant to 7 antimicrobials. Most isolates were resistant to 7–9 antimicrobials, and the broadest resistance profile was resistant to 12 antimicrobials (Figure 3C). For cefquinome, the inhibition zone diameters for the tested *K. pneumoniae* isolates ranged from 0 to 23.6 mm (Table S2). In total, 88.2% (90/102) of the isolates were MDR, defined as resistance to three or more classes of antimicrobials.

The biofilm formation assay revealed that 81.4% (83/102) of the *K. pneumoniae* isolates were biofilm-forming isolates. The proportions of strong, moderate, weak, and non-biofilm-forming isolates were 13.7% (14/102), 56.9% (58/102), 10.8% (11/102), and 18.6% (19/102), respectively.

**Figure 4:**
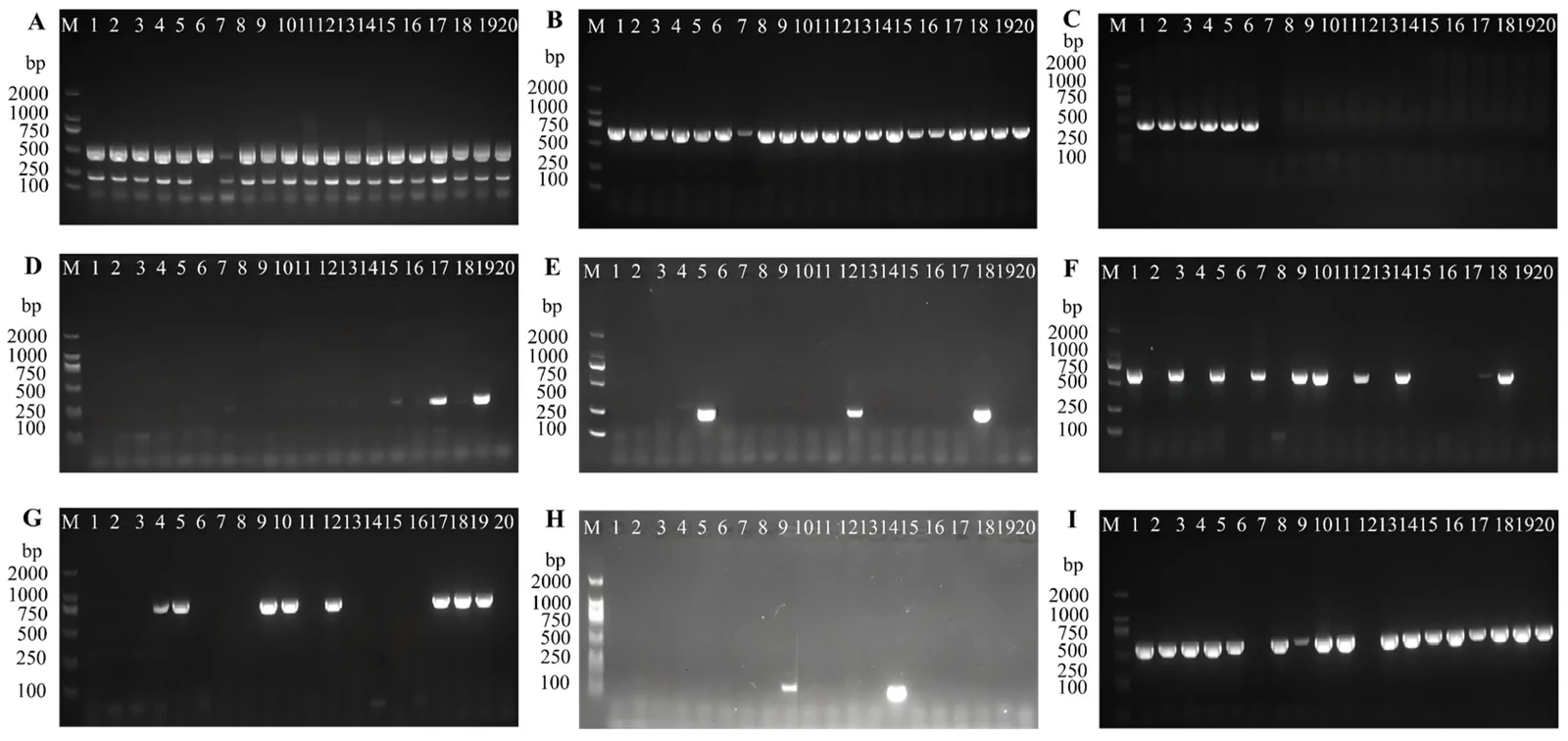
Agarose gel electrophoresis of PCR products for virulence genes. M: DL2000 DNA Ladder; (**A**): *luxS*, *mrkD*; (**B**): *wabG*; (**C**): *rmpA*; (**D**): *fimH*; (**E**): *iroB*; (**F**): *iucA*; (**G**): *ybtA*; (**H**): *wcaG*; (**I**): *uge*.

**Figure 5:**
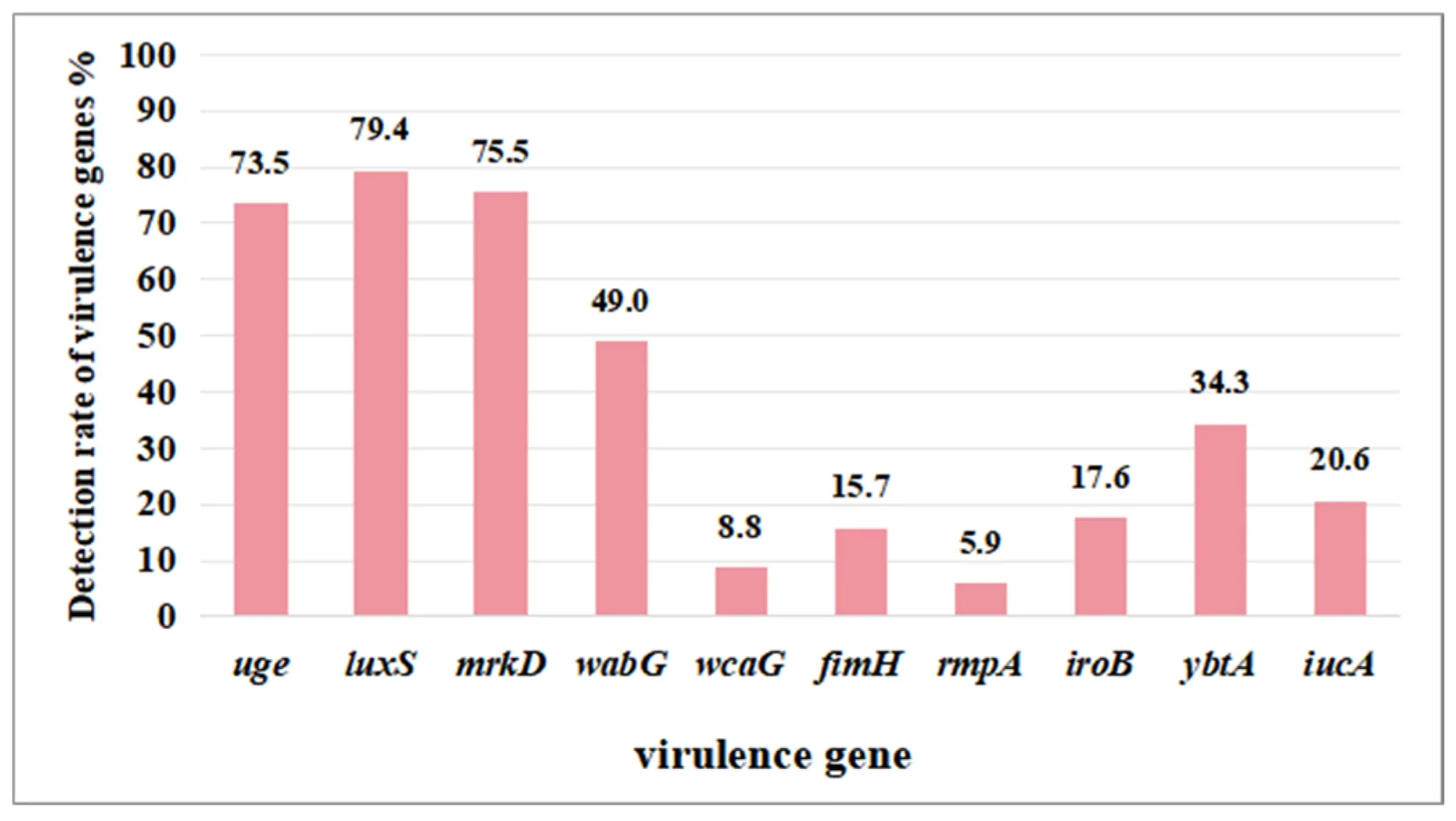
Detection rates of virulence genes.

The virulence gene detection revealed that *uge*, *mrkD*, and *luxS* had high detection rates (73.5–79.4%) among the isolates, followed by *iucA*, *ybtA*, and *wabG* (20.6–49.0%). In contrast, *rmpA*, *wcaG*, *fimH*, and *iroB* were detected at low rates (5.9–17.6%) (Figure 4 and Figure 5).

The association analysis of the hypermucoviscous phenotype with virulence factors, biofilm formation, and antimicrobial resistance revealed that all 16 hypermucoviscous isolates were MDR and exhibited varying levels of biofilm formation. Among them, 4 isolates rried both *rmpA* and *iucA* (Table 1).

**Figure 6:**
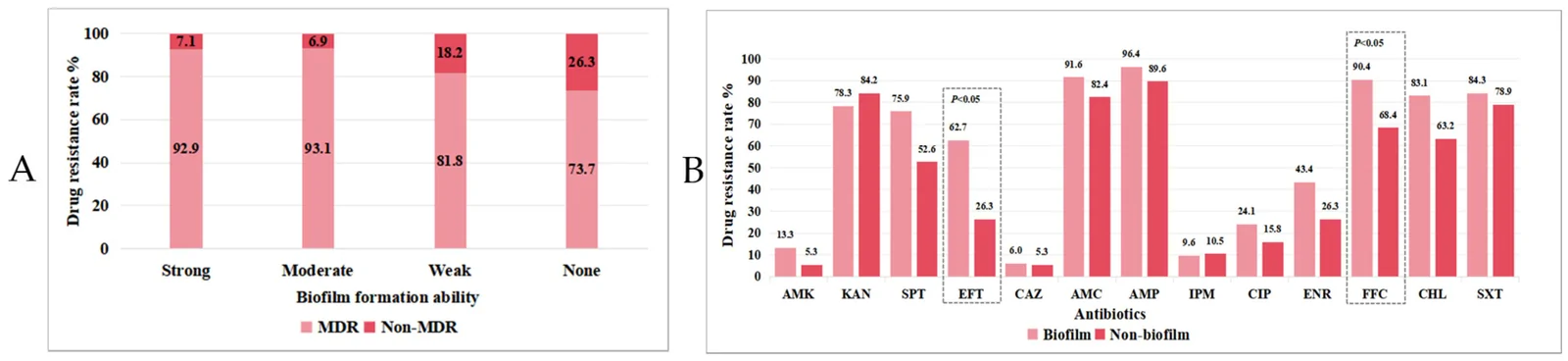
Association analysis between biofilm formation and antimicrobial resistance. (**A**): MDR and non-MDR rates among isolates with different biofilm-forming abilities. (**B**): Resistance rates of biofilm-forming and non-biofilm-forming isolates to antimicrobials.

**Figure 7:**
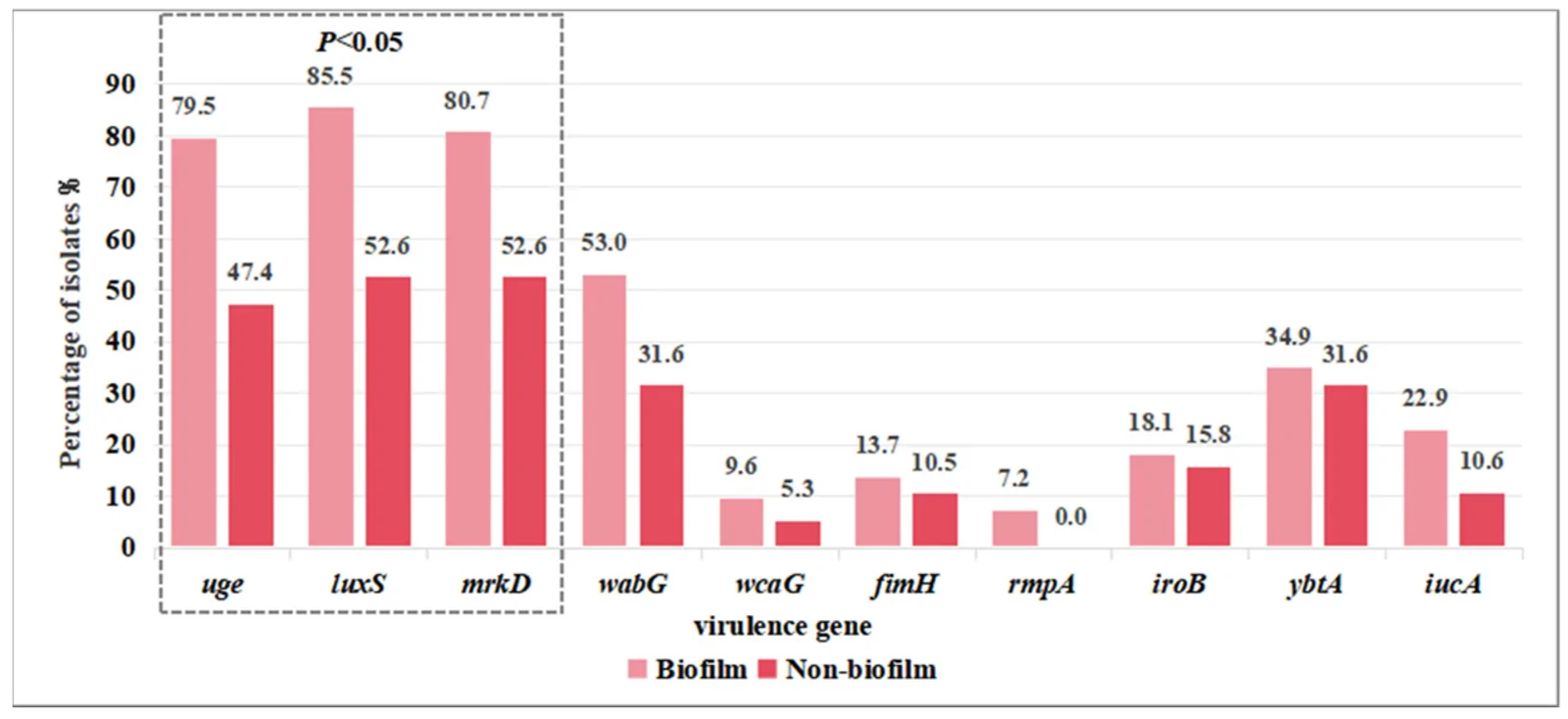
Detection rates of virulence factors between biofilm-forming and non-biofilm-forming isolates.

The association analysis of biofilm formation with antimicrobial resistance and virulence factors revealed that the proportion of MDR *K. pneumoniae* isolates was higher than that of non-MDR isolates regardless of their biofilm-forming status. However, the MDR rates of isolates with strong or moderate biofilm-forming ability were higher than those of isolates with weak or no biofilm-forming ability (Figure 6A). In addition, biofilm-forming isolates exhibited higher resistance rates to antimicrobials. Compared with non-biofilm-forming isolates, the resistance rates of biofilm-forming isolates to ceftiofur and florfenicol were significantly higher (*p* < 0.05) (Figure 6B). The detection rates of all tested virulence genes in biofilm-forming isolates were higher than those in non-biofilm-forming isolates. Among them, the detection rates of *luxS*, *mrkD*, and *uge* genes were significantly increased (*p* < 0.05) (Figure 7).

## 3. Discussion

### 3.1. Association Analysis of the Hypermucoviscous Phenotype with Virulence Factors, Biofilm Formation, and Antimicrobial Resistance

In our study, 102 chicken-derived *K. pneumoniae* isolates were identified through Gram staining and MALDI-TOF MS. The pathogenicity of *K. pneumoniae* primarily stems from its diverse virulence factors. Recently, the combined detection of the mucoid regulator genes *rmpA*/*rmpA2* and siderophore synthesis genes has been employed to identify hypervirulent *K. pneumoniae* (hvKp) [14]. Previous studies have shown that hvKp strains can produce the siderophore aerobactin, which is encoded by *iucA*, which enhances iron uptake and bacterial growth. More than 90% of hvKp isolates have been reported to carry *iucA*, making it a good marker for hypervirulence [15]. Rao et al. used molecular methods to detect *rmpA* and *iucA* to distinguish hvKp from classical *K. pneumoniae* (cKp) [14]. PCR detection of virulence genes in 102 *K. pneumoniae* isolates showed that the detection rates of *rmpA* and *iucA* were 5.9% and 20.6%, respectively, and only 4 isolates carried both genes, suggesting that only a limited number of chicken-derived isolates in Henan Province carried the hypervirulence-associated markers tested in this study. However, the string test results showed that 16 isolates exhibited a hypermucoviscous phenotype, indicating that the hypermucoviscous phenotype is not necessarily associated with the hypervirulence gene. Nguyen et al. revealed that the formation of the hypermucoviscous phenotype involves at least two independent pathways [16]. One pathway is driven by the *rmpA*/*rmpADC* operon located on virulence plasmids, whereas the other results from spontaneous mutations in the chromosomally encoded capsule export protein Wzc alters the physical structure of the capsular polysaccharide chains, thereby allowing the hypermucoviscous phenotype to arise even in the absence of virulence genes. Russo et al. demonstrated that, compared with the detection of virulence genes, the string test shows lower accuracy, sensitivity, and specificity in the identification of hvKp [17].

*K. pneumoniae* biofilm formation depends on type 3 fimbriae encoded by the *mrkABCDF* operon, which promote biofilm development by mediating the initial adhesion of bacteria to a range of surfaces and bacterial aggregation [16]. In contrast, the hypermucoviscous phenotype is mediated by the transcription factor *rmpA*, which enhances capsular polysaccharide accumulation by activating the expression of *rmpD* and *rmpC* within the *rmp* operon, leading to the formation of a thick capsule. This capsule may impede bacterial adherence to host cells. As initial adhesion is a critical step in biofilm formation, previous studies have suggested that the hypermucoviscous phenotype may inhibit biofilm formation [18,19]. However, in our study, all 16 hypermucoviscous isolates exhibited biofilm-forming ability. These findings suggest that hypermucoviscosity and biofilm formation may coexist. A clinical study by Hyun et al. provided important support for our findings [20]. The study analyzed 414 clinical isolates of *K. pneumoniae* and found that *rmpA*-positive hypermucoviscous isolates derived from liver abscesses exhibited significantly stronger biofilm-forming ability than *rmpA*-negative isolates. The authors further suggested that these isolates may harbor mutations in the *mrkABCDF* operon or alterations in regulatory factors such as CRP-cAMP and IscR, which may reduce the inhibitory effect of the hypermucoviscous phenotype on type 3 fimbriae. Consequently, these isolates may simultaneously possess biofilm-forming ability and the hypermucoviscous phenotype. These findings are consistent with the results of our study.

Notably, four *rmpA*-positive hypermucoviscous isolates in our study exhibited strong biofilm-forming ability, whereas the remaining 12 *rmpA*-negative hypermucoviscous isolates showed moderate or weak biofilm formation. These findings suggest that hypermucoviscosity arising from different regulatory mechanisms may exert distinct effects on biofilm formation. However, the number of hypermucoviscous isolates included in our study was relatively limited. Future studies are required to expand the sample size and to further elucidate the regulatory relationship between the hypermucoviscousy phenotype and biofilm formation.

### 3.2. Association Analysis Between Biofilm Formation and Virulence Factors

Biofilm formation was another important mechanism that enhanced the pathogenicity of *K. pneumoniae* [21]. It forms a physical barrier that protects bacteria from host immune clearance, thereby increasing bacterial survival and colonization [22]. During biofilm formation in *K. pneumoniae*, multiple virulence factors are involved in the regulation of different stages, among which *luxS*, *mrkD*, and *uge* were considered important regulatory factors [23]. Previous studies have shown that *luxS* was involved in quorum sensing regulation and played an important role in the formation and stability of biofilm structures; *mrkD* encoded the type 3 fimbrial adhesin and played a key role in the initial adhesion of bacteria to a range of surfaces; whereas *uge* was involved in the synthesis of LPS and contributed to maintaining bacterial cell surface structure and biofilm stability [23]. Further studies have demonstrated that when these genes were deleted, the integrity of bacterial biofilm structures, adhesion ability, and bacterial aggregation capacity were affected to varying degrees. Taken together, *luxS*, *mrkD*, and *uge* were involved in the process of biofilm formation in *K. pneumoniae* through different mechanisms [24,25,26,27]. In our study, 81.4% of the isolates were biofilm-forming, indicating that biofilm formation is common among chicken-derived *K. pneumoniae* isolates in this region. The detection results showed that the prevalence of *luxS*, *mrkD*, and *uge* exceeded 70%. Compared with non-biofilm-forming isolates, these genes were detected at significantly higher frequencies in biofilm-forming isolates. These findings suggest that these virulence factors were significantly associated with biofilm formation. Similar associations have also been reported. Khalefa et al. found that the detection rate of the *uge* ranged from 50.0% to 80.0% in *K. pneumoniae* isolates derived from horses and wild birds, and it was significantly associated with biofilm formation [28]. Daehre et al. detected the *mrkD* in all biofilm-forming *K. pneumoniae* isolates from broilers [29]. Kang et al. reported that the detection rate of the *uge* reached 93.1% in biofilm-forming *K. pneumoniae* isolates derived from bovine mastitis in Korea [30]. Odewale et al. reported that the *uge* showed the highest detection rate (68.0%) among virulence factors in human-derived *K. pneumoniae* isolates, and its prevalence reached 90.8% in biofilm-forming strains [21]. Taken together, these studies indicated that the *luxS*, *mrkD*, and *uge* were widely distributed among *K. pneumoniae* isolates from different hosts and were closely associated with biofilm formation. Currently, studies investigating the association between virulence factors and biofilm formation in chicken-derived *K. pneumoniae* isolates remain limited. The results of our study provided new epidemiological data for elucidating the relationship between virulence factors and biofilm formation in *K. pneumoniae*.

### 3.3. Association Analysis Between Biofilm Formation and Antimicrobial Resistance

Frequent use of antimicrobials in poultry production has accelerated the emergence of bacterial resistance. In our study, *K. pneumoniae* isolates from chickens showed nearly complete resistance to ampicillin, which was consistent with most previous reports. This might be due to the intrinsic resistance of *K. pneumoniae* to ampicillin [31,32]. The resistance rates of *K. pneumoniae* isolates to ceftiofur was 55.9%, which were consistent with those reported by Kot and Witeska [33]. In addition, *K. pneumoniae* isolates exhibited relatively high resistance rates to florfenicol, kanamycin, spectinomycin, sulfamethoxazole–trimethoprim (>50%), which were consistent with the trends reported in previous studies [34,35]. These antimicrobials have been commonly used in poultry production, and their frequent use may have contributed to the emergence of antimicrobial resistance [36,37]. Although *K. pneumoniae* isolates from chickens exhibited high resistance, they remained highly susceptible to imipenem and meropenem, which was consistent with the finding reported by Ammar et al., who observed a susceptibility rate of 82% to imipenem [38]. This difference might be attributed to regional disparities in antimicrobial use, infection control practices, or sample representativeness.

Based on the resistance characteristics of the *K. pneumoniae* isolates described above, our study further analyzed the association between biofilm formation and antimicrobial resistance. Biofilms protect pathogens from host immune responses and antimicrobial killing through mechanisms such as extracellular matrix barriers, efflux pump activation, and induction of persister cells, thereby enhancing bacterial resistance and survival [39,40,41]. Our study showed that 88.2% of the *K. pneumoniae* isolates exhibited multidrug-resistant phenotypes. A statistically significant association was observed between multidrug resistance and biofilm-forming ability, with isolates exhibiting stronger biofilm formation showing higher MDR rates [42,43]. These results were generally consistent with previous studies conducted on *K. pneumoniae* isolates from different sources and hosts. These studies reported a close association between biofilm formation and antimicrobial resistance in *K. pneumoniae* isolates from humans [44], cattle [45], pigs [46], and poultry [47]. Strong biofilm-forming strains usually exhibited higher levels of antimicrobial resistance.

Notably, our study found that, compared with non-biofilm-forming strains, *K. pneumoniae* isolates capable of biofilm formation exhibited significantly higher resistance rates to ceftiofur and florfenicol. This indicates that resistance to these two antimicrobials was closely associated with biofilm formation. Ceftiofur is commonly used third-generation cephalosporins, whereas florfenicol is a broad-spectrum amphenicol antimicrobial. In China, ceftiofur is legally approved for use in poultry and have been widely used for the treatment of bacterial infections in clinical practice [48]. Due to their frequent use, continuous selective pressure may have been generated. Meanwhile, biofilms could hinder the diffusion of antimicrobials to a certain extent, thereby promoting the gradual accumulation of resistance and resulting in higher resistance levels in biofilm-forming strains. Therefore, during the treatment of infections caused by biofilm-positive strains, the therapeutic efficacy of these antimicrobials might have been affected, and antimicrobial use strategies should be rationally optimized according to resistance characteristics.

This study has several inherent limitations. First, due to the lack of genomic typing and comprehensive genetic analyses, we were unable to fully assess the clonal relatedness among the isolates; therefore, we cannot completely rule out the possibility that the dissemination of a few dominant clones may have influenced some of the observed phenotypic associations. Second, this study only examined a limited set of virulence genes, and the potential roles and functions of other virulence factors and resistance genes remain to be further explored. Third, although PCR-based detection can confirm the presence of target genes, it does not reflect their transcriptional activity or functional expression; therefore, future experiments such as gene knockout and complementation assays would be helpful in clarifying the causal roles of specific genes in biofilm formation. Notwithstanding the above limitations, the present phenotypic data provide reliable and valuable baseline evidence for regional antimicrobial resistance surveillance. Given the currently scarce research data on chicken-derived Klebsiella pneumoniae in Henan Province, our core findings remain valid and informative. These deficiencies also point out clear directions for future research. Subsequent studies integrating whole-genome characterization, systematic resistance and virulence gene profiling, and in-depth functional verification with expanded sample scales will help further validate and extend the results of this work.

## 4. Materials and Methods

### 4.1. Biological Sample Source

A total of 102 *K. pneumoniae* isolates were recovered from routine diagnostic samples of liver and lung tissues from dead chickens submitted by farms across Henan Province between 2023 and 2025. Only one isolate was retained per submission batch to ensure that no multiple isolates originated from the same flock or the same outbreak event. For details, see Supplementary Table S1.

### 4.2. Isolation and Identification

Liver and lung tissue samples were surface-sterilized by brief flaming with an alcohol-soaked swab. Approximately 1 g of internal tissue was excised. The tissue sample was then streaked onto nutrient agar (Aobox Biotechnology, Beijing, China) plates using the three-zone streaking method and incubated at 37 °C for 24 h. Following incubation, all single colonies were picked and subcultured on blood agar plates (Beijing Tiangrun Biotechnology Co., Ltd., Beijing, China) for further purification. The selected colonies were identified by Gram staining. Three single colonies were then spotted onto the MALDI target plate. Immediately, 1 μL of 70% formic acid (Macklin, Shanghai, China) was added to cover each spot, followed by 1 μL of α-cyano-4-hydroxycinnamic acid (Thermo Fisher Scientific, Waltham, MA, USA) matrix solution. After the matrix had dried, the protein fingerprint spectra of the isolates were acquired using a Bruker MALDI Biotyper mass spectrometer (Bruker Daltonics, Bremen, Germany). The spectra were processed with the Biotyper software and compared with the reference spectra in the Bruker MBT Clinical Library (DB8468_2969). Identification results were interpreted as follows: scores of 2.000–3.000 indicated species-level identification; scores of 1.700–1.999 indicated genus-level identification; scores of 0–1.699 were considered unreliable.

### 4.3. String Test for Hypermucoviscosity

The strains were seeded on blood agar plates and incubated at 37 °C for 24 h. A standard bacteriologic loop was used to stretch a mucoviscous string from the colony. Hypermucoviscosity was defined by the formation of viscous strings longer than 5 mm. A human clinical *K. pneumoniae* isolate with a confirmed hypermucoviscous phenotype, previously characterized in our laboratory, was used as the positive control for the string test [23].

### 4.4. Biofilm Formation

A single colony was picked and inoculated into TSB broth (BRT, Beijing, China), then incubated at 37 °C with shaking overnight. The turbidity of the overnight culture was adjusted to a 0.5 McFarland standard (Solarbio, Beijing, China) using TSB broth. The adjusted bacterial suspension was diluted 1:100 with fresh TSB. Then 1 mL of the diluted bacterial suspension was added to each well of a 24-well plate (Thermo Fisher Scientific, Waltham, MA, USA), with three replicate wells per isolate, and TSB medium alone was used as a negative control. The plate was incubated at 37 °C for 24 h. After incubation, the wells were washed three times with sterile deionized water. Then 1 mL of methanol (Macklin, Shanghai, China) was added to each well and fixed for 15 min. The methanol was removed and the wells were washed three times with sterile deionized water. Next, 1 mL of 0.01% (*w*/*v*) crystal violet solution (Solarbio, Beijing, China) was added to each well and stained for 15 min. The stain was aspirated and the wells were washed three times. Finally, 1 mL of 33% (*v*/*v*) glacial acetic acid (Fuyu, Tianjin, China) was added to each well to destain. After mixing, 200 µL from each well was transferred to a 96-well plate and the optical density at 570 nm (OD_570_) was measured using a Synergy/HTX spectrophotometer (BioTek Instruments, Inc., Winooski, VT, USA). The cut-off optical density (ODc) was defined as twice the mean OD value of the blank (negative control) wells. Based on the relationship of each sample’s OD to ODc, biofilm-forming ability was classified as follows: strong biofilm producer (OD > 4 × ODc), moderate biofilm producer (2 × ODc < OD ≤ 4 × ODc), weak biofilm producer (ODc < OD ≤ 2 × ODc), and non-biofilm producer (OD ≤ ODc) [49,50].

### 4.5. Antimicrobial Resistance

The bacterial resistance profile was determined via the agar diffusion method using antibiotic-impregnated disks, in accordance with the Clinical and Laboratory Standards Institute guidelines CLSI M100, CLSI VET01S and T/ZNZ 099-2021. The antimicrobials tested were amikacin (AMK, 30 µg/dis, R ≤ 14 mm), kanamycin (KAN, 30 µg/disk, R ≤ 13 mm), spectinomycin (SPT, 100 µg/disk, R ≤ 10 mm), ceftiofur (EFT, 30 µg/disk, R ≤ 17 mm), ceftazidime (CAZ, 30 µg/disk, R ≤ 17 mm), cefquinome (CFQ, 30 µg/disk), amoxicillin–clavulanic acid (AMC, 20/10 µg/disk, R ≤ 13 mm), ampicillin (AMP, 10 µg/disk, R ≤ 13 mm), imipenem (IPM, 10 µg/disk, R ≤ 19 mm), meropenem (MEM, 10 µg/disk, R ≤ 19 mm), ciprofloxacin (CIP, 5 µg/disk, R ≤ 21 mm), enrofloxacin (ENR, 5 µg/disk, R ≤ 16 mm), florfenicol (FFC, 30 µg/disk, R ≤ 14 mm), chloramphenicol (CHL, 30 µg/disk, R ≤ 12 mm), trimethoprim-sulfamethoxazole (SXT, 25 µg/disk, R ≤ 10 mm) (all purchased from BKMAM, Changde, China). *K. pneumoniae* ATCC 700603 was used as the quality control strain.

According to the definition by Magiorakos et al., multidrug resistance (MDR) was defined as acquired resistance to ≥3 antimicrobial categories. Since *K. pneumoniae* is intrinsically resistant to aminopenicillins due to a chromosomally encoded β-lactamase, ampicillin was excluded from the MDR classification in this study. The MDR phenotype was determined based on the acquired resistance categories to the remaining antimicrobial agents tested [51].

### 4.6. Virulence Factors

The virulence factors were identified by PCR. DNA extraction was performed according to the method described by Ouyang et al. [52]. The amplification mixture consisted of PCR buffer (10×), 2.5 mM dNTP Mixture, 5 U/L of Taq DNA polymerase (all amplification mixture were purchased from TaKaRa, Kusatsu, Japan), 1 μM of each primer (Sangon, Shanghai, China), and autoclaved ultrapure water to a final volume of 20 µL.

The virulence genes searched were *luxS*, *uge*, *mrkD*, *fimH*, *iucA*, *iroB*, *rmpA*, *ybtA*, *wabG*, and *wcaG*. Table 2 presents the functions of the studied virulence genes and the corresponding references. Detailed primer information, including primer sequences, amplicon sizes, and amplification conditions, is provided in Supplementary Material Table S3.

The amplification products were separated by electrophoresis on a 2% agarose gel (Solarbio, Beijing, China) and examined after staining with GelBlue (TaKaRa, Kusatsu, Japan). The molecular weight marker used was the 2000 bp DNA ladder (TaKaRa, Kusatsu, Japan).

### 4.7. Statistical Analysis

Data analysis was conducted using the GraphPad Prism version 8.0.2 (GraphPad Software, San Diego, CA, USA). Associations between variables were assessed using the chi-square test, and *p* < 0.05 was considered statistically significant.

## 5. Conclusions

Chicken-derived *K. pneumoniae* isolates in Henan carried diverse virulence factors, and MDR was widely observed. Although the detection rates of hypervirulence genes (such as *rmpA* and *iucA*) were relatively low, the high prevalence of *luxS*, *mrkD*, and *uge*, along with the finding that 81.4% of the isolates were capable of biofilm formation, suggested that these isolates still exhibited pathogenic potential. Association analysis showed that biofilm formation was positively correlated with both virulence genes and antimicrobial resistance. Moreover, biofilm-forming strains exhibited significantly higher resistance rates to ceftiofur and florfenicol, as well as higher detection rates of *luxS*, *mrkD*, and *uge* than non-biofilm-forming strains. These results suggested that *luxS*, *mrkD*, and *uge* might play important roles in *K. pneumoniae* biofilm formation and were closely associated with enhanced antimicrobial resistance. This study provided important theoretical and data support for the effective prevention and control of chicken-derived *K. pneumoniae* infections in this region, as well as for rational clinical antimicrobial use and the development of anti-biofilm strategies.

## Figures and Tables

**Table 1:** Resistance and virulence factors of hypermucoviscosity strains in association with biofilm production.

Strain	Virulence Gene	Biofilm
1	*luxS*, *mrkD*, *uge*, *wabG*, *fimH*, *iucA*	Moderate
2	*luxS*, *mrkD*, *uge*, *wabG*, *wcaG*, *iucA*, *ybtA*	Moderate
3	*luxS*, *mrkD*, *uge*, *wabG*, *rmpA*, *fimH*, *iucA*, *ybtA*	Strong
4	*luxS*, *mrkD*, *uge*, *wabG*, *wcaG*, *rmpA*, *fimH*, *iroB*, *iucA*, *ybtA*	Strong
5	*mrkD*, *luxS*, *uge*, *wabG*, *fimH*, *iroB*, *ybtA*	Moderate
6	*luxS*, *mrkD*, *wabG*, *wcaG*, *fimH*, *ybtA*	Weak
7	*luxS*, *mrkD*, *uge*, *wabG*, *wcaG*, *fimH*, *ybtA*	Moderate
8	*luxS*, *mrkD*, *uge*, *wcaG*, *fimH*, *iucA*, *ybtA*	Moderate
9	*luxS*, *mrkD*, *uge*, *wabG*, *fimH*	Moderate
10	*luxS*, *mrkD*, *uge*, *wabG*, *wcaG*, *ybtA*	Moderate
11	*luxS*, *mrkD*, *uge*, *wabG*, *rmpA*, *fimH*, *iucA*, *ybtA*	Strong
12	*luxS*, *mrkD*, *uge*, *wabG*, *wcaG*, *fimH*	Moderate
13	*mrkD*, *uge*, *wabG*, *wcaG*, *fimH*, *ybtA*	Weak
14	*luxS*, *mrkD*, *uge*, *wabG*, *fimH*, *ybtA*	Moderate
15	*luxS*, *mrkD*, *uge*, *wabG*, *rmpA*, *fimH*, *iucA*, *ybtA*	Strong
16	*luxS*, *markD*, *uge*, *wabG*, *fimH*, *iucA*, *ybtA*	Moderate

**Table 2:** Genes, function and references used for the identification of virulence factors in *K. pneumoniae*.

Target Gene	Function	References
*luxS*	autoinducer-2 synthase	[28]
*uge*	uridine diphosphate galacturonate 4-epimerase	[28]
*mrkD*	type 3 adhesin	[28]
*fimH*	type 1 adhesin	[53]
*iucA*	aerobactin siderophore synthesis protein	[54]
*iroB*	salmochelin siderophore glycosyltransferase	[54]
*rmpA*	regulator of the mucoid phenotype A	[23]
*ybtA*	transcriptional regulator of yersiniabactin synthesis	[55]
*wabG*	lipopolysaccharide core biosynthesis protein	[55]
*wcaG*	GDP-fucose synthetase	[55]

## Data Availability

The data presented in this study are available in the Supplementary Materials accompanying this article.

## Supplementary Materials

The following supporting information can be downloaded at https://www.mdpi.com/article/10.3390/antibiotics15080767/s1, Table S1: Source, isolation site, and isolation time of the examined *K. pneumoniae* isolates. Table S2: Biofilm-forming ability, Susceptibility category and virulence genes of *K. pneumoniae* isolates. Table S3: Primer sequences used for PCR amplification of virulence factors in *K. pneumoniae*.
