# Supplementary material for: Associations of Biofilm Capacity with Antimicrobial Resistance and Virulence Genes in Klebsiella pneumoniae from Chickens in Henan, China, 2023–2025
Source: Antibiotics (Basel). 2026 Aug 10;15(8):767. doi: 10.3390/antibiotics15080767 (PMC13509524; doi:10.3390/antibiotics15080767)
Supplement: Supplementary file 1 [file antibiotics-15-00767-s001.zip › antibiotics-4428154-supplementary.pdf]

Table S3. Primer sequences used for PCR amplification of virulence factors in *K. pneumoniae*

| Target gene | Primer sequence (5'-3') | Amplicon size (bp) | Amplification conditions |
|---|---|---|---|
| *luxS* | F: GCCGTTGTTAGATAGTTTCACAG<br>R: CAGTTCGTCGTTGCTGTTGATG | 447 | Initial denaturation of 2 min at 95 °C followed by 35 cycles, denaturation at 95 °C for 20 s, annealing at 53 °C for 30 s, extension at 72 °C for 45 s, and final extension at 72 °C for 7 min |
| *uge* | F: TCT TCA CGC CTT CCT TCA CT<br>R: GAT CAT CCG GTC TCC CTG TA | 535 | |
| *mrkD* | F: CCACCAACTATTCCCTCGAA<br>R: ATGGAACCCACATCGACATT | 226 | |
| *fimH* | F：CCATAACGATTACCCAGAA<br>R：GACATCACGAGCAGAAGC | 373 | Initial denaturation of 2 min at 95 °C followed by 35 cycles, denaturation at 95 °C for 30 s, annealing at 54 °C for 30 s, extension at 72 °C for 30 s, and final extension at 72 °C for 7 min |
| *iucA* | F: GCTTATTTCTCCCCAACCC<br>R: TCAGCCCTTTAGCGACAAG | 583 | Initial denaturation of 2 min at 95 °C followed by 35 cycles, denaturation at 95 °C for 30 s, annealing at 59 °C for 50 s, extension at 72 °C for 30 s, and final extension at 72 °C for 7 min |
| *iroB* | F: ATCTCATCATCTACCCTCCGCTC<br>R: GGTTCGCCGTCGTTTTCAA | 235 | Initial denaturation of 2 min at 95 °C followed by 35 cycles, denaturation at 95 °C for 30 s, annealing at 59 °C for 30 s, extension at 72 °C for 30 s, and final extension at 72 °C for 7 min |
| *rmpA* | F:CATAAGAGTATTGGTTGACAG<br>R.:CTTGCATGAGCCATCTTTCA | 461 | Initial denaturation of 2 min at 95 °C followed by 30 cycles, denaturation at 95 °C for 30 s, annealing at 60 °C for 45 s, extension at 72 °C for 30 s, and final extension at 72 °C for 7 min |
| *ybtA* | F:ATGACGGAGTCACCGCAAAC<br>R: TTACATCACGCGTTTAAAGG | 960 | Initial denaturation of 2 min at 95 °C followed by 30 cycles, denaturation at 95 °C for 30 s, annealing at 55 °C for 50 s, extension at 72 °C for 30 s, and final extension at 72 °C for 7 min |
| *wabG* | F: CGGACTGGCAGATCCATATC<br>R: ACCATCGGCCATTTGATAGA | 683 | Initial denaturation of 2 min at 95 °C followed by 30 cycles, denaturation at 95 °C for 30 s, annealing at 53 °C for 40 s, extension at 72 °C for 30 s, and final extension at 72 °C for 7 min |
| *wcaG* | F:GGTTGGKTCAGCAATCGTA<br>R:ACTATTCCGCCAACTTTTGC | 169 | Initial denaturation of 2 min at 95 °C followed by 30 cycles, denaturation at 95 °C for 30 s, annealing at 58 °C for 30 s, extension at 72 °C for 30 s, and final extension at 72 °C for 7 min |
